# Complete Genome Sequence of *Geobacter anodireducens* SD-1^T^, a Salt-Tolerant Exoelectrogenic Microbe in Bioelectrochemical Systems

**DOI:** 10.1128/genomeA.00415-16

**Published:** 2016-06-02

**Authors:** Dan Sun, Shaoan Cheng, Aijie Wang, Fangliang Huang, Wenzong Liu, Xue Xia

**Affiliations:** a Ocean College, Zhejiang University, Zhoushan, China; b Department of Energy Engineering, State Key Laboratory of Clean Energy Utilization, Zhejiang University, Hangzhou, China; c Key Laboratory of Environmental Biotechnology, Research Center for Eco-Environmental Sciences, Beijing, China; d College of Life Sciences, Zhejiang University, Hangzhou, China

## Abstract

Strain SD-1 is the type strain of the species *Geobacter anodireducens*, which was originally isolated from a microbial fuel cell reactor in the United States. The characteristic of this bacterium is its high electrochemical activity. Here, we report the fully assembled genome and plasmid sequence of *G. anodireducens* SD-1^T^.

## GENOME ANNOUNCEMENT

*Geobacter anodireducens* SD-1 (=CGMCC 1.12536 = KCTC 4672), the type strain of the species *G. anodireducens*, was first isolated from a mature anode biofilm in a bioelectrochemical system (BES) based on its current generation capability (1, 2). Compared to the type exoelectrogenic microbes of *Geobacter sulfurreducens* PCA and *Geobacter metallireducens* GS-15, strain SD-1 showed higher electrochemical activity in BESs (1 – 3). The extracellular electron transfer (EET) of anode microbes has been studied intensely in the past decade (4). However, our understanding of how microorganisms transfer electrons to/from electrodes is still incomplete, and only a few different microbes have been genetically studied. To facilitate genetic studies of the EET mechanism, we assembled and annotated the genome of *G. anodireducens* SD-1^T^.

The *G. anodireducens* SD-1^T^ genome was sequenced using ABI PGM. A 3-kb mate-pair library and a 200-bp library were prepared from sheared genomic DNA using the 5500 SOLiD mate-paired library kit and Ion Xpress Plus fragment library kit, respectively. Subread filtering from 2 libraries yielded 1.4 Gbp of sequence. The sequences were checked, trimmed, and assembled in Genomics Workbench 6.0 (CLC bio, Aarhus, Denmark). The complete genome of SD-1^T^ was obtained, consisting of a circular chromosome of 3,555,507 bp in length (average coverage, 261×) and a circular plasmid of 110,507 bp (average coverage, 160×), with G+C contents of 61% and 52.17%, respectively. The NCBI staff used the Prokaryotic Genome Annotation Pipeline (PGAP) (http://ncbi.nlm.nih.gov/genomes/static/Pipeline.html) to complete the annotation; there are 3,407 predicted genes in the genome, including 2,802 protein-coding genes, 547 pseudogenes, 48 tRNA-coding genes, 6 rRNA-coding genes, 4 noncoding RNA (ncRNA), and 1 clustered regularly interspaced short palindromic repeat (CRISPR) array.

### Nucleotide sequence accession numbers.

The *G. anodireducens* SD-1^T^ genome sequence and annotation data are available from GenBank under accession numbers CP014963 and CP014964.
